# Evaluation of Dried Blood Spots and Oral Fluids as Alternatives to Serum for Human Papillomavirus Antibody Surveillance

**DOI:** 10.1128/mSphere.00043-18

**Published:** 2018-05-09

**Authors:** Karly S. Louie, Jama Dalel, Caroline Reuter, Sara L. Bissett, Michelle Kleeman, Lesley Ashdown-Barr, Rawinder Banwait, Anna Godi, Peter Sasieni, Simon Beddows

**Affiliations:** a Centre for Cancer Prevention, Wolfson Institute of Preventive Medicine, Queen Mary University of London, London, United Kingdom; b Virus Reference Department, Public Health England, London, United Kingdom; Albert Einstein College of Medicine

**Keywords:** HPV vaccine, human papillomavirus, antibodies, dried blood spot, oral fluids, surveillance

## Abstract

Human papillomavirus (HPV) vaccination elicits high-titer genotype-specific antibody responses that are associated with a reduced risk of cervical disease caused by vaccine-incorporated genotypes. Our objective was to evaluate dried blood spots (DBSs) and oral mucosal transudate (OMT) as alternative samples to serum to confirm HPV vaccine antibody status. A study was carried out to evaluate the feasibility of detecting HPV16 and HPV18 antibodies in OMT, DBSs, and sera among women who self-reported being unvaccinated or fully vaccinated with the HPV vaccine. Serum had the highest sensitivity (100%) for detection of antibodies against both HPV16 and HPV18 but the lowest specificity, due to the detection of natural infection antibodies in 16% of unvaccinated women. Conversely, DBSs and OMT had lower sensitivity (96% and 82%, respectively) but high specificity (98%). We confirmed that these antibodies were functional (i.e., neutralizing) and that their detection was quantitatively reproducible and well correlated between sample types when normalized to IgG content. DBSs and OMT are appropriate alternative sample types for HPV vaccine surveillance. These alternative sample types warrant consideration for the purposes of cervical screening, diagnosis, and management, but more work will be needed to establish the stringent parameters required for such application.

**IMPORTANCE** Human papillomavirus (HPV) is the causative agent of cervical and other anogenital cancers. HPV vaccination, primarily targeted at young girls before the age of sexual debut, is starting to demonstrate population-level declines in HPV infection and early disease associated with vaccine-incorporated genotypes. Monitoring young women for vaccine-specific antibody is important for vaccine surveillance and may be useful as an adjunct test within a cervical screening context. We evaluated serum, dried blood spots, and oral fluid as potential samples for such applications and report robust measures of diagnostic accuracy. This is the first time a direct comparison of alternative sample types has been made between vaccinated and unvaccinated women for the detection and quantitation of HPV antibodies.

## Abstract (precis)

Human papillomavirus (HPV) is the causative agent of cervical and other anogenital cancers. HPV vaccination, primarily targeted at young girls before the age of sexual debut, is starting to demonstrate population-level declines in HPV infection and early disease associated with vaccine-incorporated genotypes. Monitoring young women for vaccine-specific antibody is important for vaccine surveillance and may be useful as an adjunct test within a cervical screening context. We evaluated serum, dried blood spots, and oral fluid as potential samples for such applications and report robust measures of diagnostic accuracy. This is the first time a direct comparison of alternative sample types has been made between vaccinated and unvaccinated women for the detection and quantitation of HPV antibodies.

## OBSERVATION

Prophylactic human papillomavirus (HPV) virus-like particle (VLP) vaccines, targeting the two most prevalent oncogenic genotypes, HPV16 and HPV18, have demonstrated 90 to 100% efficacy against HPV16/18-associated cervical lesions in vaccine trials (1). National HPV immunization programs have been introduced in many countries worldwide (2) and are starting to provide evidence for a high degree of vaccine effectiveness in these populations (3). Vaccine-induced antibodies are thought to be the primary mediators of protection (4), and serology may inform vaccine impact surveillance and vaccine coverage and potentially be useful as an adjunct test for cervical screening, diagnosis, and management.

Serum is generally considered the “gold standard” sample to measure surrogates of vaccine-induced protective immunity (5). Dried blood spots (DBSs) and oral fluids, specifically oral mucosal transudate (OMT), offer attractive alternatives to serum antibody testing and have already been found to be appropriate tools for the antibody surveillance and/or diagnosis of HIV (6), hepatitis C (7), and measles (8). Finger prick DBS samples are easy to collect in clinical and nonclinical settings and do not require specialist equipment for processing and storage. OMT can be self-collected and may be more acceptable than venipuncture or finger prick (9). Limited data are available evaluating DBSs (10) and OMT (11) as alternative samples for HPV antibody detection. We carried out a study to evaluate the diagnostic accuracy of serum, DBS, and OMT samples for the determination of HPV vaccination status.

Study characteristics of vaccinated (*n =* 50) and unvaccinated (*n =* 103) women taking part are shown in Table 1. Vaccinated women were younger and less sexually experienced than unvaccinated women, although the ages at first sex were the same in both groups. Unvaccinated women had more lifetime sexual partners than vaccinated women, had a history of sexually transmitted infection(s), and were more likely to be positive for high-risk HPV in the vaginal swab sample, including vaccine genotypes HPV16 and -18. Among vaccinated women, the estimated time since vaccination at time of the study was 4 years.

**TABLE 1:** Study population characteristics

Parameter^a^	Result for:		*P* value^b^
	Vaccinated	Unvaccinated	
Total *n*	50	103	
Age, median yr (IQR)	21 (20–22)	26 (25–28)	<0.001
Time since vaccination, median yr (IQR)^c^	4 (4–4)		
Ever had sex, *n* (%)	40 (80)	103 (100)	<0.001
Age at first sex, median yr (IQR)	17 (16–18)	17 (16–18)	0.260
Lifetime sex partners, median *n* (IQR)	3 (2–7)	10 (5–15)	<0.001
Ever had an STI, *n* (%)	4 (8)	48 (47)	<0.001
Provided genital swab, *n* (%)	34 (68)	74 (72)	0.624
High-risk HPV positive, *n* (%)^d^	11 (32)	51 (69)	0.001
HPV16/18 positive, *n* (%)^d^	2 (6)	17 (23)	0.030

a HPV, human papillomavirus; IQR, interquartile range; STI, sexually transmitted infection.

b Differences between proportions were assessed using the chi-square test, while differences between continuous variables were assessed using the Mann-Whitney *U* test.

c Estimated time since vaccination at time of the study based upon year of survey − HPV cohort year.

d HPV DNA positivity in optional vaginal sample that was provided by 108 subjects (34 vaccinated and 74 unvaccinated).

All vaccinated women were seropositive for antibodies against both HPV16 and HPV18 VLPs compared to 16% of unvaccinated women (Table 2). IgG recovered from DBSs (median, 170 µg/ml; interquartile range [IQR], 122 to 244 µg/ml) and OMT (median, 28 µg/ml; IQR, 21 to 44 µg/ml) samples were as indicated. Dual positivity for antibodies against HPV16 and HPV18 was substantially lower for unvaccinated than vaccinated women for DBSs (2% versus 96%) and OMT (2% versus 82%). As expected, median HPV16 antibody binding titers were higher than HPV18 titers, and this was reflected in all sample types (see Table S1 in the supplemental material).

10.1128/mSphere.00043-18.1 TABLE S1  HPV16 and HPV18 binding antibody titers and antibody levels in serum, DBS, and OMT samples. Download TABLE S1, PDF file, 0.3 MB. © Crown copyright 2018. 2018 Crown This content is distributed under the terms of the Creative Commons Attribution 4.0 International license.

**TABLE 2:** Antibody positivity against HPV16 and/or HPV18 VLPs for serum, DBS, and OMT samples^a^

Sample type and antigen	Vaccinated		Unvaccinated		Diagnostic accuracy					
	Antibody positive, *n*/total (%)	Median antibody titer (IQR)	Antibody positive, *n*/total (%)	Median antibody titer (IQR)	Threshold titer	Sensitivity, %	Specificity, %	PPV, %	NPV, %	AUC
Serum										
VLP16	48/48 (100)	3,515 (1,909–6,630)	33/103 (32)	25 (25–141)	609	98	95	90	99	0.965
VLP18	48/48 (100)	1,747 (913–2,296)	27/103 (26)	25 (25–78)	330	100	94	89	100	0.971
VLP16/18	48/48 (100)	NA	16/103 (16)	NA	NA	98	98	96	99	0.980
DBS										
VLP16	46/46 (100)	54.4 (21.4–82.0)	11/103 (11)	2.5 (2.5–2.5)	11.4	98	92	85	99	0.950
VLP18	44/46 (96)	44.4 (18.4–83.8)	4/103 (4)	2.5 (2.5–2.5)	5.4	96	96	92	98	0.959
VLP16/18	44/46 (96)	NA	2/103 (2)	NA	NA	94	98	96	97	0.958
OMT										
VLP16	48/50 (96)	5.6 (3.6–16.3)	4/100 (4)	1.0 (1.0–1.0)	2.1	92	96	92	96	0.940
VLP18	41/50 (82)	4.5 (3.0–16.9)	2/100 (2)	1.0 (1.0–1.0)	2.4	80	98	95	91	0.890
VLP16/18	41/50 (82)	NA	2/100 (2)	NA	NA	80	98	95	91	0.890

a Antibody positivity is based upon the following limits of detection: serum, 50; DBSs, 5; and OMT, 2. For the analysis presented here, samples negative for a particular target were assigned a censored titer of half that of the limit of detection. Diagnostic accuracy (sensitivity, specificity, positive predictive value [PPV], negative predictive value [NPV], and area under the curve [AUC]) was estimated using a threshold titer derived from receiver operator characteristic (ROC) analysis. DBS, dried blood spot; IQR, interquartile range; NA, not applicable; OMT, oral mucosal transudate; VLP, virus-like particle.

**FIG 1:**
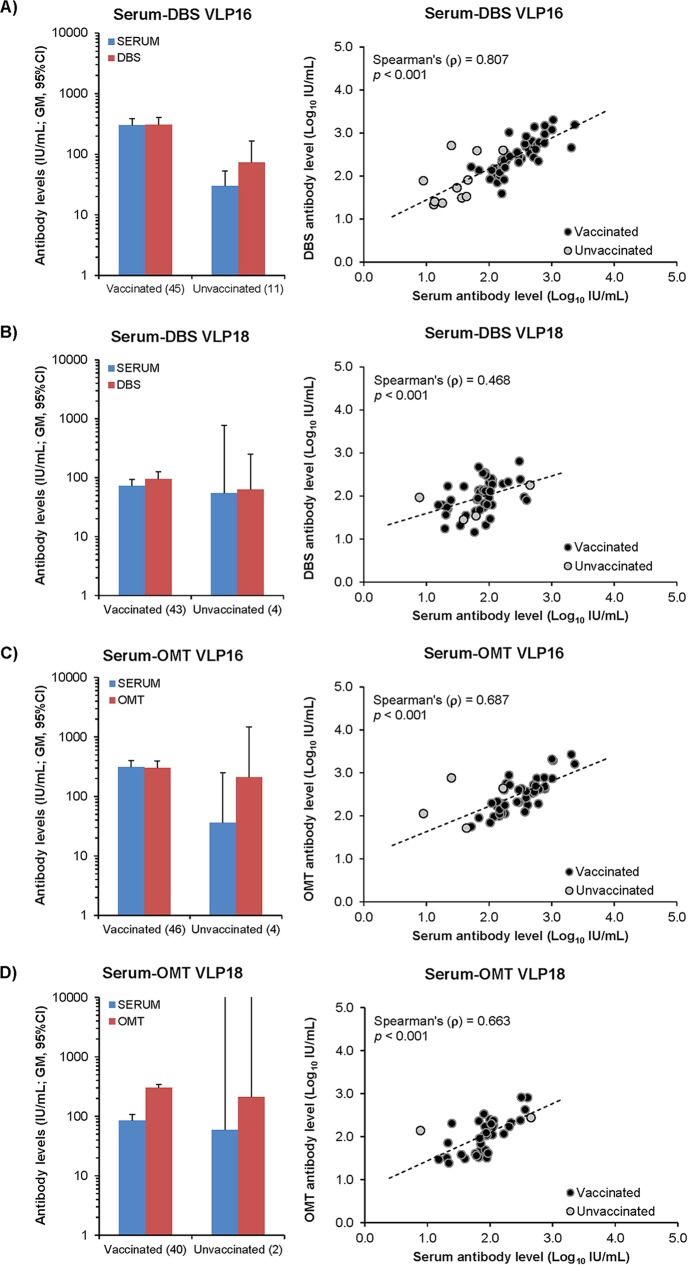
Comparison between antibody levels in serum and DBSs or OMT. Antibody levels in serum were compared to normalized antibody levels in DBSs (A and B) or OMT (C and D) for HPV16 (A and C) and HPV18 (B and D) antigens. The left panels present geometric mean antibody levels (with 95% CI) for vaccinated and unvaccinated individuals (with *n* in parentheses) positive for both sample types. The right panels present correlation plots between serum and alternative sample type for each antigen.

HPV antibody-positive samples demonstrate a close relationship between antibody levels (IU per milliliter) in serum compared with DBSs or OMT when normalized by sample IgG concentration (Fig. 1). Serum, DBS, and OMT samples from a subset of vaccinees (*n =* 8) were tested in the pseudovirus neutralization assay against HPV16 and HPV18 and demonstrated similar titers between the assays (*r*^*2*^ = 0.961) (see Table S2 in the supplemental material).

10.1128/mSphere.00043-18.2 TABLE S2  HPV16 and HPV18 neutralizing and binding antibody titers for serum, DBS, and OMT samples. Download TABLE S2, PDF file, 0.1 MB. © Crown copyright 2018. 2018 Crown This content is distributed under the terms of the Creative Commons Attribution 4.0 International license.

The areas under the curve (AUC) for dual HPV16 and HPV18 VLP positivity demonstrated high discriminative accuracy for the determination of vaccine status using serum (AUC, 0.980; 95% confidence interval [CI], 0.955 to 1.000), DBSs (AUC, 0.958; 95% CI, 0.919 to 0.996), and OMT (AUC, 0.890; 95% CI, 0.832 to 0.948) (Table 2). The highest specificity was obtained for dual antibody detection in DBSs and OMT (98% each), but with reduced sensitivity compared to detection of HPV16 antibodies alone.

To our knowledge, this is the first study to evaluate the accuracy of serum, DBSs, and OMT in parallel to discriminate between HPV-vaccinated and unvaccinated women based upon detection of both HPV16 and HPV18 antibodies. Although serum HPV antibody levels are 10 to 100 times higher in vaccinated than unvaccinated women (12, 13), antibody titers derived from natural infection overlap the lower boundary of titers generated by vaccination (14), leading to a slightly reduced specificity for determination of vaccine status for this sample type.

Conversely, the detection rate for natural infection antibodies was much lower in DBS and OMT samples (ca. 2%), due to sample (DBS) or host (OMT) dilution effects, while retaining a high rate of antibody positivity, and therefore specificity, for vaccinees. With the widespread introduction of HPV vaccines, it is important to correctly identify women who have been vaccinated for the purposes of robust vaccination impact monitoring, informing future changes to vaccination policy and potentially future cervical screening strategies. We believe it is more important to have a test with high specificity than high sensitivity so that very few women will be wrongly classified as having been vaccinated. A previous study demonstrated good agreement for paired serum and DBS samples but was limited to an unvaccinated population (10). This study also used DBSs obtained by venipuncture rather than finger prick, as in our study, which likely reflects more accurately the performance of DBSs in real-life settings. Although both DBSs and OMT have high discriminative accuracies, OMT may be more convenient and acceptable and simpler for processing and testing (6).

We found better discriminative accuracy for OMT than an early clinical trial of the monovalent HPV16 vaccine for determining HPV16 antibody status (11). We also found that antibody titers for all sample types derived from the VLP enzyme-linked immunosorbent assay (ELISA) correlate well with the neutralization assay, which is in agreement with a recent study that detected both HPV16 and HPV18 neutralizing antibodies in OMT of vaccinees (15).

This study had several strengths in terms of the systematic methodological approach in this research. Simultaneous samples of venous blood, DBSs, and OMT were taken. We were able to demonstrate the efficiency of recovery of intact immunoglobulins, although it was lower for OMT than for DBSs. We also demonstrated that these IgGs were functional (i.e., neutralizing) and quantitatively related to the levels found in serum. We developed receiver operating characteristic (ROC) curves to determine the sensitivity and specificity of DBSs, OMT, and sera for HPV16, HPV18, and dual HPV16/18 antibody cutoff points using the self-reported vaccination status (none versus all 3 doses) as the reference variable.

This study had some shortcomings. The populations were not randomly selected and may not represent the ideal target populations for determining HPV vaccination status for the purposes of vaccine impact surveillance or for women undergoing cervical screening. The vaccination status of individual participants was self-reported. It is unlikely, however, that women would have been assigned incorrectly to the vaccinated or unvaccinated group, based upon their characteristics and antibody titers, but there is a possibility that some of the vaccinated women may have incorrectly recollected whether they received all three doses or not.

Our findings suggest that DBSs and OMT are appropriate alternative sample types for HPV vaccine surveillance where a slightly reduced sensitivity may not be a problem, but that more work is needed to establish whether such sample types could be useful for cervical screening, diagnosis, and management, similar to that of HIV, hepatitis C virus (HCV), and measles (6–8). As HPV vaccination becomes widely adopted, prevention of cervical cancer will require a combined strategy of vaccination and screening. Potentially, among vaccinated women, the cervical screening age may be raised and screening intervals may be extended. However, these changes in cervical screening strategies may not be made without an effective way of identifying a woman’s overall risk of developing cervical cancer. Aside from primary testing for HPV during cervical screening, an appropriate test to confirm HPV vaccination status will be needed to adequately quantify a woman’s overall risk.

### Study population.

Vaccinated and unvaccinated participants were recruited from two different study settings. The United Kingdom introduced a routine immunization program in 2008 using the bivalent HPV vaccine targeting girls aged 12 to 13 and a catch-up program for girls up to age 17 for the first 2 years of the program, resulting in high vaccination coverage of the routine cohort (>80% for all three doses) and lower coverage in the catch-up cohort (50%) (14). At the time of the study, female university students would have been vaccinated during the catch-up program. For convenience of sampling, women ≥18 years of age who self-reported receiving three doses of the HPV vaccine in the United Kingdom and provided informed consent were recruited from Queen Mary University of London (QMUL). To ensure adequate sampling of unvaccinated women who would have a measurable rate of HPV antibody positivity due to natural infection, a different setting where women were slightly older and displayed riskier sexual behavior was used. Sexually active women 18 to 30 years of age who self-reported being unvaccinated and attending a sexual health clinic were recruited.

Ethical approval was obtained from the UK National Research Ethics Committee (13/LO/1729 and 15/LO/1093).

### Data and sample collection and processing.

Participants self-completed a questionnaire to collect data on demographics and sexual behavior and provided three specimen samples (venous blood, DBSs, and OMT) for anonymous HPV serology testing. An optional self-collected vaginal sample was also provided for anonymous HPV DNA testing.

Whole blood (4 ml) was obtained and allowed to clot for 30 to 60 min at room temperature and then centrifuged for 10 min at 1,300 × *g*. Finger prick blood samples were collected to saturate five 1.2-cm-diameter DBS circles (GE Healthcare Bio-Sciences, Pittsburgh, PA) and allowed to dry at room temperature for 4 to 24 h. OMTs were collected using an Oracol saliva swab device (Malvern Medical Developments, Ltd., Worcester, United Kingdom). Participants were instructed to saturate the swab for 60 s. Phosphate-buffered saline (PBS; 1 ml) containing 0.2% Tween 20 and 10% fetal calf serum was subsequently added to the saturated swab. Verbal and written instructions were given for the self-collected vaginal swab sample (Digene female swab specimen collection kit; Qiagen, West Sussex, United Kingdom). Collected samples were stored on cold packs and transported back to the Molecular Epidemiology Laboratory (MEL) at QMUL on the same day of collection and stored at between 2 and 8°C for next day processing.

Within 24 h of collection, OMT was extracted from the Oracol device by centrifugation (1,200 rpm for 10 min), and 250 µl of the supernatant was removed for analysis. A Harris Micro-punch (2.0-mm diameter) was used to punch the DBS card 9 times to obtain a total of 28 mm^2^. Elution of DBS was achieved using PBS (200 µl) containing 0.05% Tween 20 and overnight incubation at 4°C with gentle shaking (600 rpm; Eppendorf Thermomixer). Processed samples were stored at −80°C until HPV serology testing was carried out at Public Health England and HPV DNA testing at MEL.

### HPV serology.

Baculovirus-expressed HPV16 and HPV18 L1 VLPs were purified on an iodixanol gradient, visualized by SDS-PAGE and negative-stain electron microscopy, and used as target antigens in an enzyme-linked immunosorbent assay (ELISA), as previously described (16). International standards for HPV16 (IS16) and HPV18 (IS18) antibodies (codes 05/134 and 10/140, respectively; National Institute for Biological Standards and Control, United Kingdom) and the high-HPV16/18 and HPV-negative plasma pools (17) were used as controls. IS16 and IS18 generated median titers against HPV16 and HPV18 of 151 (IQR, 132 to 162; *n =* 13) and 219 (IQR, 182 to 270; *n =* 12), respectively. The high-HPV16/18 reagent generated titers against HPV16 and HPV18 of 56,153 (IQR, 30,182 to 73,404; *n =* 13) and 17,023 (IQR, 8,837 to 19,985; *n =* 12). Antibody levels were assigned by dividing the study serum (or control plasma) titer by the titer of the appropriate IS and multiplying by the IS assigned number of IU per milliliter (10 or 16 IU/ml for IS16 and IS18, respectively). Thus, the high-HPV16/18 reagent generated antibody levels of 3,719 IU/ml (IQR, 1,999 to 4,861; *n =* 13) and 1,246 IU/ml (IQR, 647 to 1,463; *n =* 12) when standardized against IS16 and IS18, respectively. Where both serum and DBS (or serum and OMT) samples were positive against a particular antigen, antibody levels were assigned to the DBS or OMT sample after first normalizing the titer using the IgG concentration of both the serum and DBS or OMT for that individual.

The HPV-negative pool was negative in all tests. For analysis purposes, individual serum (limit of detection [LOD], 50), DBS (LOD, 5), or OMT (LOD, 2) samples that were negative for VLP binding at the lowest dilution used were assigned a value of half that level. For sample repeatability purposes, serum, DBS, and OMT samples from a small number of individuals (*n =* 12) were retested with a resulting Pearson’s *r*^*2*^ value of 0.986. A subset of samples was also tested in the pseudovirus neutralization assay using HPV16 and HPV18 pseudoviruses as targets, as previously described (18).

IgG levels in serum, DBS, and OMT samples were determined using an IgG capture ELISA as previously described, with minor modifications (16). Detection was resolved using a goat anti-human alkaline phosphatase-conjugated antibody and NADPH-based amplification, as for the VLP ELISA, with NOR-01 (human IgG standard; Nordic-MUbio, The Netherlands) used as an internal calibrator. The median Pearson’s *r*^*2*^ value of the standards was 0.989 (IQR, 0.979 to 0.995; *n =* 16).

### HPV DNA detection and genotyping.

DNA was extracted from vaginal cells using the QIAamp DNA minikit on a Qiacube instrument (Qiagen, West Sussex, United Kingdom) following the manufacturer’s instructions, except for an overnight incubation at 56°C. DNA was stored at between −15 and −25°C before testing.

Samples were tested using the DNA ELISA kit HPV SPF10 version 1 (Labo Biomedical Products, Rijswikj, The Netherlands), which detects 40 HPV types. HPV-positive samples were then genotyped using a reverse hybridization line probe assay identifying 25 HPV types (HPV6, -11, -16, -18, -31, -33, -34, -35, -39, -40, -42, -43, -44, -45, -51, -52, -53, -54, -56, -58, -59, -66, -68, -70, and -74) (RHA kit HPV SPF10 LiPA25 version 1; Labo Biomedical Products, Rijswijk, the Netherlands) following the manufacturer’s instructions.

### Statistical analysis.

Continuous variables were expressed as the median and IQR, and categorical variables were described as frequencies. To compare study characteristics between vaccinated and unvaccinated women, continuous variables were compared using the Mann-Whitney *U* test and categorical variables by the chi-square test. Titer differences between paired assay data were tested by Wilcoxon’s signed-rank test. Receiver operating characteristic (ROC) curve analyses were performed, and individual HPV16 and HPV18 optimal titer cutoffs were determined using Youden’s index. These cutoffs were then used to evaluate the discriminative accuracy of each sample type (sensitivity, specificity, positive predictive value [PPV], negative predictive value [NPV], and area under the curve [AUC]). All analyses were performed using Stata version 13 (StataCorp, College Station, TX).
