# Supplementary material for: Evaluation of Dried Blood Spots and Oral Fluids as Alternatives to Serum for Human Papillomavirus Antibody Surveillance
Source: mSphere. 2018 May 9;3(3):e00043-18. doi: 10.1128/mSphere.00043-18 (PMC5956145; doi:10.1128/mSphere.00043-18)
Supplement: TABLE S1 [file sph003182545st1.pdf]

Table S1. HPV16 and HPV18 binding antibody titers and antibody levels in serum, DBS and OMT samples

Group	SERUM	IgG	VLP16 Titer	IU/mL	Log10 IU/mL	VLP18 Titer	IU/mL	Log10 IU/mL	DBS	IgG	VLP16 Titer	IU/mL Eq	Log10 IU/mLEq	VLP18 Titer	IU/mL Eq	Log10 IU/mLEq	OMT	IgG	VLP16 Titer	IU/mL Eq	Log10 IU/mLEq	VLP18 Titer	IU/mL Eq	Log10 IU/mLEq
Vaccinated	001A	7,554	9,190	784	2.9	3,611	167	2.2	001B	110	324.6	1467	3.2	120.7	189	2.3	001C	23	19.7	422	2.6	15.6	116	2.1
Vaccinated																	002C	16	3.4			<2		
Vaccinated	003A	11,382	4,862	415	2.6	1,603	74	1.9	003B	105	83.2	597	2.8	56.0	139	2.1	003C	15	3.5	179	2.3	2.9	51	1.7
Vaccinated	004A	9,566	2,124	181	2.3	1,487	69	1.8	004B	122	67.0	347	2.5	75.1	135	2.1	004A	18	3.1	112	2.0	3.7	45	1.7
Vaccinated	005A	9,562	1,705	145	2.2	2,469	114	2.1	005B	109	22.0	127	2.1	116.9	235	2.4	005C	18	2.8	98	2.0	15.3	182	2.3
Vaccinated	006A	15,624	1,409	120	2.1	866	40	1.6	006B	159	22.5	146	2.2	73.6	165	2.2	006C	42	3.9	96	2.0	3.6	31	1.5
Vaccinated	007A	10,262	6,011	513	2.7	2,184	101	2.0	007B	119	76.6	436	2.6	101.2	199	2.3	007C	46	24.1	352	2.5	32.9	167	2.2
Vaccinated	008A	18,164	5,765	492	2.7	1,882	87	1.9	008B	120	64.0	641	2.8	99.7	345	2.5	008C	17	4.7	333	2.5	4.3	105	2.0
Vaccinated	009A	21,859	810	69	1.8	394	18	1.3	009B	186	17.3	135	2.1	22.8	61	1.8	009C	39	2.4	89	2.0	<2		
Vaccinated	010A	21,043	9,137	779	2.9	6,649	307	2.5	010B	267	178.5	928	3.0	351.5	632	2.8	010C	32	10.8	469	2.7	15.9	239	2.4
Vaccinated	011A	16,097	4,609	393	2.6	1,398	64	1.8	011B	118	53.3	481	2.7	26.2	82	1.9	011C	23	5.5	260	2.4	<2		
Vaccinated	012A	17,384	1,940	166	2.2	1,551	72	1.9	012B	70	14.1	230	2.4	21.3	120	2.1	012C	16	<2			<2		
Vaccinated	013A	12,976	1,959	167	2.2	1,432	66	1.8									013C	44	9.8	191	2.3	4.9	33	1.5
Vaccinated	014A	10,979	4,410	376	2.6	2,365	109	2.0	014B	120	91.2	552	2.7	120.9	253	2.4	014C	46	7.8	123	2.1	20.3	110	2.0
Vaccinated	015A	10,174	12,443	1061	3.0	1,750	81	1.9	015B	145	432.5	2008	3.3	85.5	137	2.1	015C	26	75.4	1917	3.3	12.1	106	2.0
Vaccinated	016A	14,167	12,074	1030	3.0	4,721	218	2.3									016C	28	21.6	734	2.9	17.9	210	2.3
Vaccinated	017A	14,141	6,883	587	2.8	4,409	203	2.3	017B	140	79.2	529	2.7	91.0	210	2.3	017C	26	11.3	406	2.6	13.7	170	2.2
Vaccinated	018A	16,170	4,611	393	2.6	1,943	90	2.0	018B	137	105.9	826	2.9	112.8	304	2.5	018C	49	17.0	368	2.6	14.4	108	2.0
Vaccinated	019A	23,458	6,839	583	2.8	2,428	112	2.0									019C	23	11.0	740	2.9	8.6	200	2.3
Vaccinated	020A	15,077	609	52	1.7	338	16	1.2	020B	119	19.3	161	2.2	21.1	61	1.8	020C	37	2.1	56	1.7	<2		
Vaccinated	021A	27,586	2,456	210	2.3	1,743	80	1.9	021B	147	82.9	1028	3.0	78.5	337	2.5	021C	35	16.8	879	2.9	18.7	337	2.5
Vaccinated	022A	20,365	5,312	453	2.7	478	22	1.3	022B	190	47.1	334	2.5	20.2	50	1.7	022C	5	<2			<2		
Vaccinated	023A	14,779	1,579	135	2.1	330	15	1.2	023B	147	15.3	102	2.0	<5			023C	27	4.7	169	2.2	2.4	29	1.5
Vaccinated	024A	9,046	1,888	161	2.2	1,270	59	1.8	024B	177	11.4	38	1.6	12.2	14	1.2	024C	23	4.3	113	2.1	4.4	40	1.6
Vaccinated	025A	7,170	1,228	105	2.0	1,934	89	2.0	025B	117	20.6	84	1.9	14.9	21	1.3	025C	22	3.2	69	1.8	4.3	32	1.5
Vaccinated	026A	11,215	27,481	2344	3.4	7,942	366	2.6	026B	135	280.0	1536	3.2	49.1	93	2.0	026C	43	93.7	1604	3.2	71.4	422	2.6
Vaccinated	027A	11,694	7,446	635	2.8	2,432	112	2.1	027B	81	63.7	605	2.8	18.8	62	1.8	027C	34	18.3	411	2.6	22.3	173	2.2
Vaccinated	028A	18,227	1,877	160	2.2	430	20	1.3	028B	131	21.1	194	2.3	5.4	17	1.2	028C	20	2.9	178	2.3	<2		
Vaccinated	029A	25,226	3,896	332	2.5	1,802	83	1.9	029B	158	23.8	251	2.4	14.8	54	1.7	029C	22	5.6	424	2.6	6.6	171	2.2
Vaccinated	030A	19,382	3,516	300	2.5	394	18	1.3	030B	110	24.0	280	2.4	<5			030C	9	2.9	400	2.6	<2		
Vaccinated	031A	21,621	1,916	163	2.2	2,160	100	2.0	031B	126	20.7	235	2.4	22.9	90	2.0	031C	34	4.0	166	2.2	17.3	250	2.4
Vaccinated	032A	9,544	6,073	518	2.7	2,273	105	2.0	032B	128	55.0	270	2.4	17.1	29	1.5	032C	23	18.9	522	2.7	20.6	196	2.3
Vaccinated	033A	12,390	7,228	617	2.8	1,828	84	1.9	033B	268	71.9	220	2.3	63.0	66	1.8	033C	21	4.9	190	2.3	3.6	49	1.7
Vaccinated	034A	11,959	3,514	300	2.5	1,321	61	1.8	034B	207	53.8	206	2.3	32.9	44	1.6	034C	51	13.4	206	2.3	6.8	36	1.6
Vaccinated	035A	10,413	9,002	768	2.9	2,474	114	2.1	035B	128	107.3	576	2.8	99.8	185	2.3	035C	40	44.2	770	2.9	39.3	237	2.4
Vaccinated	036A	9,109	1,595	136	2.1	1,550	71	1.9	036B	175	20.1	69	1.8	39.6	47	1.7	036C	11	3.9	214	2.3	3.6	68	1.8
Vaccinated	037A	17,063	1,300	111	2.0	466	21	1.3	037B	133	15.9	135	2.1	18.3	54	1.7	037C	22	3.8	196	2.3	4.0	71	1.9
Vaccinated	038A	13,682	3,314	283	2.5	446	21	1.3	038B	157	61.6	355	2.5	18.1	36	1.6	038C	28	6.4	209	2.3	2.8	32	1.5
Vaccinated	039A	23,621	2,211	189	2.3	1,442	67	1.8	039B	342	49.3	225	2.4	55.7	88	1.9	039C	41	14.9	562	2.7	17.6	230	2.4
Vaccinated	040A	20,291	1,746	149	2.2	928	43	1.6	040B	288	21.5	100	2.0	21.7	35	1.5	040C	25	2.1	110	2.0	<2		
Vaccinated	041A	19,212	2,043	174	2.2	536	25	1.4	041B	172	24.5	234	2.4	15.5	80	1.9	041C	9	3.0	446	2.6	4.0	202	2.3
Vaccinated	042A	11,794	6,271	535	2.7	1,486	69	1.8	042B	215	292.7	1369	3.1	184.5	467	2.7	042C	10	4.4	358	2.6	3.2	91	2.0
Vaccinated	043A	12,023	24,243	2068	3.3	8,661	400	2.6	043B	142	62.9	453	2.7	20.2	79	1.9	043C	27	89.5	2667	3.4	78.3	807	2.9
Vaccinated	044A	12,035	1,732	148	2.2	480	22	1.3	044B	202	23.4	119	2.1	60.8	168	2.2	044C	41	7.2	139	2.1	3.6	24	1.4
Vaccinated	045A	13,139	2,118	181	2.3	2,028	94	2.0	045B	153	21.3	156	2.2	15.8	62	1.8	045C	20	4.1	176	2.2	2.8	41	1.6
Vaccinated	046A	11,351	6,560	560	2.7	1,888	87	1.9	046B	226	96.6	413	2.6	55.8	129	2.1	046C	78	51.3	492	2.7	37.1	123	2.1
Vaccinated	047A	9,937	11,969	1021	3.0	6,892	318	2.5	047B	247	342.9	1176	3.1	130.2	242	2.4	047C	14	44.1	2073	3.3	50.5	820	2.9
Vaccinated									048B	260	60.6			66.2			048C	6	3.9			4.6		
Vaccinated	049A	17,038	2,049	175	2.2	759	35	1.5	049B	348	19.4	81	1.9	9.1	20	1.3	049C	37	13.0	396	2.6	3.6	38	1.6
Vaccinated	050A	12,981	2,516	215	2.3	2,273	105	2.0	050B	241	61.3	282	2.4	51.1	127	2.1	050C	22	13.2	524	2.7	14.4	198	2.3
Unvaccinated	001A	12,201	<50			<50			001B	305	<5			<5			001C	29	<2			<2		
Unvaccinated	002A	10,386	<50			426	39	1.6	002B	327	<5			9	28	1.4	002C	49	<2			<2		
Unvaccinated	003A	8,263	75	5	0.7	<50			003B	100	<5			<5			003C	23	<2			<2		
Unvaccinated	004A	8,657	<50			<50			004B	199	<5			<5			004C	55	<2			<2		
Unvaccinated	005A	5,409	163	10	1.0	<50			005B	79	<5			<5			005C	11	<2			<2		
Unvaccinated	006A	5,665	<50			<50			006B	174	<5			<5			006C	18	<2			<2		
Unvaccinated	007A	8,279	208	13	1.1	111	10	1.0	007B	166	<5			<5			007C	24	<2			<2		
Unvaccinated	008A	10,128	139	9	0.9	<50			008B	232	<5			<5			008C	18	<2			<2		
Unvaccinated	009A	11,506	<50			<50			009B	157	<5			<5			009C	19	<2			<2		
Unvaccinated	010A	14,194	<50			<50			010B	431	<5			<5			010C	94	<2			<2		
Unvaccinated	011A	12,722	<50			<50			011B	121	<5			<5			011C	22	<2			<2		
Unvaccinated	012A	10,994	<50			<50			012B	210	<5			<5			012C	69	<2			<2		
Unvaccinated	013A	7,929	<50			162	15	1.2	013B	62	<5			<5			013C	52	<2			<2		
Unvaccinated	014A	9,048	585	37	1.6	173	16	1.2	014B	165	11	31	1.5	<5			014C	67	<2			<2		
Unvaccinated	015A	6,067	503	32	1.5	<50			015B	239	<5			<5			015C	82	<2			<2		
Unvaccinated	016A	8,362	<50			101	9	1.0	016B	273	<5			<5			016C	28	<2			<2		
Unvaccinated	017A	9,424	<50			<50			017B	190	<5			<5			017C	89	<2			<2		
Unvaccinated	018A	14,097	<50			<50			018B	324	<5			<5			018C	57	<2			<2		
Unvaccinated	019A	13,812	<50			<50			019B	255	<5			<5			019C	36	<2			<2		
Unvaccinated	020A	12,726	<50			<50			020B	309	<5			<5			020C	48	<2			<2		
Unvaccinated	021A	15,331	<50			183	17	1.2	021B	294	<5			<5			021C	10	<2			<2		
Unvaccinated	022A	6,085	695	44	1.6	<50			022B	178	20	33	1.5	<5			022C	41	7	52	1.7	<2		
Unvaccinated	023A	6,373	<50			<50			023B	144	<5			<5			023C	18	<2			<2		
Unvaccinated	024A	10,624	<50			<50			024B	303	<5			<5			024C	37	<2			<2		
Unvaccinated	025A	8,997	<50			<50			025B	136	<5			<5			025C	22	<2			<2		
Unvaccinated	026A	9,426	<50			<50			026B	149	<5			<5			026C	11	<2			<2		
Unvaccinated	027A	9,683	167	11	1.0	<50			027B	175	<5			<5			027C	22	<2			<2		
Unvaccinated	028A	9,015	<50			<50			028B	203	<5			<5			028C	20	<2			<2		
Unvaccinated	029A	8,862	<50			<50			029B	158	<5			<5			029C	15	<2			<2		
Unvaccinated	030A	10,912	<50			119	11	1.0	030B	247	<5			<5			030C	53	<2			<2		
Unvaccinated	031A	10,336	<50			<50			031B	250	<5			<5			031C	60	<2			<2		
Unvaccinated	032A	9,244	178	11	1.1	143	13	1.1	032B	107	<5			<5			032C	44	<2			<2		
Unvaccinated	033A	9,315	<50			<50			033B	219	<5			<5			033C	22	<2			<2		
Unvaccinated	034A	10,636	197	12	1.1	72	7	0.8	034B	164	<5			<5			034C	39	<2			<2		
Unvaccinated	035A	9,105	<50			<50			035B	208	<5			<5			035C	31	<2			<2		
Unvaccinated	036A	8,869	253	16	1.2	165	15	1.2	036B	241	<5			<5			036C	34	<2			<2		
Unvaccinated	037A	9,190	<50			135	13	1.1	037B	283	<5			<5			037C	18	<2			<2		
Unvaccinated	038A	9,609	211	13	1.1	<50			038B	343	<5			<5			038C	124	<2			<2		
Unvaccinated	039A	11,822	<50			<50			039B	348	<5			<5			039C	80	<2			<2		
Unvaccinated	040A	9,892	<50			<50			040B	440	<5			<5			040C	54	<2			<2		
Unvaccinated	041A	9,758	129	8	0.9	178	16	1.2	041B	207	<5			<5			041C	62	<2			<2		
Unvaccinated	042A	7,930	<50			<50			042B	186	<5			<5			042C	41	<2			<2		
Unvaccinated	043A	10,070	238	15	1.2	<50			043B	287	<5			<5			043C	62	<2			<2		
Unvaccinated	044A	9,520	<50			<50			044B	202	<5			<5			044C	38	<2			<2		
Unvaccinated	045A	6,490	<50			<50			045B	157	<5			<5			045C	18	<2			<2		
Unvaccinated	046A	9,029	<50			<50			046B	140	<5			<5			046C	55	<2			<2		
Unvaccinated	047A	10,534	<50			<50			047B	108	<5			<5			047C	52	<2			<2		
Unvaccinated	048A	9,486	<50			<50			048B	113	<5			<5			048C	26	<2			<2		
Unvaccinated	049A	9,725	<50			<50			049B	247	<5			<5			049C	73	<2			<2		
Unvaccinated	050A	9,086	<50			<50			050B	297	<5			<5			050C	43	<2			<2		
Unvaccinated	051A	8,622	173	11	1.0	<50			051B	200	<5			<5			051C	50	<2			<2		

Unvaccinated	052A	8,633	<50			214	20	1.3	052B	244	<5			<5			052C	75	<2			<2		
Unvaccinated	053A	6,023	<50			<50			053B	219	<5			<5			053C	48	<2			<2		
Unvaccinated	054A	9,303	286	18	1.3	<50			054B	206	10	23	1.4	<5			054C	21	<2			<2		
Unvaccinated	055A	9,624	730	46	1.7	<50			055B	180	30	80	1.9	<5										
Unvaccinated	056A	11,310	<50			672	62	1.8	056B	335	<5			10	34	1.5								
Unvaccinated	057A	9,077	209	13	1.1	<50			057B	118	6	21	1.3	<5										
Unvaccinated	058A	7,666	<50			<50			058B	211	<5			<5			058C	21	<2			<2		
Unvaccinated	059A	10,328	57	4	0.6	<50			059B	315	<5			<5			059C	49	<2			<2		
Unvaccinated	060A	8,919	<50			<50			060B	297	<5			<5			060C	38	<2			<2		
Unvaccinated	061A	9,527	<50			<50			061B	299	<5			<5			061C	60	<2			<2		
Unvaccinated	062A	6,516	<50			<50			062B	183	<5			<5			062C	21	<2			<2		
Unvaccinated	063A	8,810	491	31	1.5	490	45	1.7	063B	109	13	52	1.7	<5			063C	35	<2			<2		
Unvaccinated	064A	9,911	<50			<50			064B	247	<5			<5			064C	52	<2			<2		
Unvaccinated	065A	5,755	55	3	0.5	140	13	1.1	065B	142	<5			<5			065C	42	<2			<2		
Unvaccinated	066A	9,410	<50			<50			066B	170	<5			<5			066C	50	<2			<2		
Unvaccinated	067A	11,182	<50			<50			067B	306	<5			<5			067C	9	<2			<2		
Unvaccinated	068A	9,238	<50			<50			068B	220	<5			<5			068C	25	<2			<2		
Unvaccinated	069A	8,793	216	14	1.1	93	9	0.9	069B	263	15	25	1.4	<5			069C	39	<2			<2		
Unvaccinated	070A	9,891	<50			<50			070B	356	<5			<5			070C	26	<2			<2		
Unvaccinated	071A	8,492	<50			<50			071B	249	<5			<5			071C	31	<2			<2		
Unvaccinated	072A	9,004	<50			139	13	1.1	072B	286	<5			<5			072C	37	<2			<2		
Unvaccinated	073A	8,557	<50			<50			073B	125	<5			<5			073C	39	<2			<2		
Unvaccinated	074A	10,498	847	53	1.7	920	85	1.9	074B	251	<5			<5			074C	43	<2			<2		
Unvaccinated	075A	11,308	143	9	1.0	84	8	0.9	075B	158	22	77	1.9	13	92	2.0	075C	42	8	113	2.1	5	138	2.1
Unvaccinated	076A	8,822	<50			<50			076B	186	<5			<5			076C	31	<2			<2		
Unvaccinated	077A	6,058	<50			<50			077B	105	<5			<5			077C	6	<2			<2		
Unvaccinated	078A	10,536	400	25	1.4	4,905	453	2.7	078B	363	352	505	2.7	61	178	2.2	078C	60	86	758	2.9	15	275	2.4
Unvaccinated	079A	6,091	58	4	0.6	120	11	1.0	079B	160	<5			<5			079C	16	<2			<2		
Unvaccinated	080A	9,858	<50			<50			080B	235	<5			<5			080C	41	<2			<2		
Unvaccinated	081A	11,640	<50			<50			081B	216	<5			<5			081C	72	<2			<2		
Unvaccinated	082A	9,590	<50			<50			082B	99	<5			<5			082C	34	<2			<2		
Unvaccinated	083A	6,754	<50			117	11	1.0	083B	82	<5			<5			083C	50	<2			<2		
Unvaccinated	084A	8,791	241	15	1.2	<50			084B	51	<5			<5			084C	5	<2			<2		
Unvaccinated	085A	9,369	<50			<50			085B	102	<5			<5			085C	16	<2			<2		
Unvaccinated	086A	8,549	139	9	0.9	<50			086B	55	<5			<5			086C	24	<2			<2		
Unvaccinated	087A	13,899	<50			<50			087B	157	<5			<5			087C	19	<2			<2		
Unvaccinated	088A	8,800	<50			<50			088B	120	<5			<5			088C	30	<2			<2		
Unvaccinated	089A	8,728	<50			<50			089B	150	<5			<5			089C	22	<2			<2		
Unvaccinated	090A	9,038	<50			<50			090B	156	<5			<5			090C	11	<2			<2		
Unvaccinated	091A	8,895	156	10	1.0	<50			091B	105	<5			<5			091C	22	<2			<2		
Unvaccinated	092A	8,765	2,663	168	2.2	379	35	1.5	092B	89	81	394	2.6	<5			092C	8	8	436	2.6	<2		
Unvaccinated	093A	10,881	<50			<50			093B	141	<5			<5			093C	51	<2			<2		
Unvaccinated	094A	9,753	<50			<50			094B	83	<5			<5			094C	10	<2			<2		
Unvaccinated	095A	9,496	<50			<50			095B	94	<5			<5			095C	15	<2			<2		
Unvaccinated	096A	10,271	<50			<50			096B	118	<5			<5			096C	27	<2			<2		
Unvaccinated	097A	11,550	1,019	64	1.8	<50			097B	106	71	386	2.6	<5			097C	26	<2			<2		
Unvaccinated	098A	10,027	<50			132	12	1.1	098B	109	<5			<5			098C	34	<2			<2		
Unvaccinated	099A	9,117	<50			<50			099B	79	<5			<5			099C	25	<2			<2		
Unvaccinated	100A	13,659	<50			<50			100B	220	<5			<5			100C	28	<2			<2		
Unvaccinated	101A	9,493	186	12	1.1	111	10	1.0	101B	132	<5			<5			101C	21	<2			<2		
Unvaccinated	102A	14,739	167	11	1.0	111	10	1.0	102B	123	<5			<5			102C	32	<2			<2		
Unvaccinated	103A	9,865	<50			<50			103B	64	<5			<5			103C	10	<2			<2		

Eq, Equivalent antibody levels (IU/mL) estimated by normalizing the antibody titer as a function of the sample IgG concentration and the serum IgG concentration for that individual
DBS, Dried Blood Spots; OMT, Oral Mucosal Transudate
