# Supplementary material for: Evaluation of Dried Blood Spots and Oral Fluids as Alternatives to Serum for Human Papillomavirus Antibody Surveillance
Source: mSphere. 2018 May 9;3(3):e00043-18. doi: 10.1128/mSphere.00043-18 (PMC5956145; doi:10.1128/mSphere.00043-18)
Supplement: TABLE S2 [file sph003182545st2.pdf]

Table S2. HPV16 and HPV18 neutralizing and binding antibody titers for serum, DBS and OMT samples

	HPV16						HPV18					
	Neutralizing antibody titer			Binding antibody titer			Neutralizing antibody titer			Binding antibody titer		
Sample	Serum	DBS	OMT	Serum	DBS	OMT	Serum	DBS	OMT	Serum	DBS	OMT
010	8,005	288	17	9,137	179	11	10,533	275	19	6,649	352	16
015	25,977	466	57	12,443	433	75	2,238	81	<10	1,750	86	12
018	9,980	234	25	4,611	106	17	7,317	207	27	1,943	113	14
026	39,939	ND	146	27,481	280	94	29,513	ND	107	7,942	49	71
035	8,548	225	71	9,002	107	44	8,330	222	49	2,474	100	39
043	49,845	143	108	24,243	63	90	26,091	141	99	8,661	20	78
046	5,383	130	47	6,560	97	51	6,923	141	42	1,888	56	37
047	20,665	489	48	11,969	343	44	12,356	305	39	6,892	130	51

DBS, Dried Blood Spots; OMT, Oral Mucosal Transudate; ND, Not Done
